# IL2RA is a prognostic indicator and correlated with immune characteristics of pancreatic ductal adenocarcinoma

**DOI:** 10.1097/MD.0000000000030966

**Published:** 2022-10-21

**Authors:** Liwen Fan, Xinyu Wang, Qing Chang, Yue Wang, Wenjie Yang, Linlin Liu

**Affiliations:** a Department of Radiotherapy, China-Japan Union Hospital of Jilin University, Changchun, Jilin Province, China; b Department of Breast Surgery, The Second Hospital of Jilin University, Changchun, Jilin Province, China; c Department of Pathogenobiology, College of Basic Medical Sciences, Jilin University, Changchun, Jilin Province, China.

**Keywords:** IL2RA, immune infiltration, pancreatic ductal adenocarcinoma, prognosis

## Abstract

Pancreatic ductal adenocarcinoma (PDAC) is a highly aggressive and incurable cancer with a dismal prognosis. In this study, we aimed to explore potential predictors for the prognosis and immunological characteristics of PDAC.

Estimation of stromal and immune cells in malignant tumors, using expression data (ESTIMATE) method was applied to calculate the immune and stromal scores of 206 PDAC samples from GSE71729. R package of “limma” was utilized to identify differentially expressed genes (DEGs). Gene ontology (GO) and Kyoto encyclopedia of genes and genomes (KEGG) enrichment analyses were conducted for functional exploration. Protein-protein interaction (PPI) network and Univariate Cox analysis were conducted to select key prognostic genes of PDAC. Gene set enrichment analysis (GSEA) was applied to investigate the roles of IL2RA in PDAC. Single sample GSEA (ssGSEA) was performed to evaluate the immunological characteristics of PDAC samples. Wilcoxon rank sum test was conducted to compare the difference of immunological characteristics of PDAC samples between low IL2RA and high IL2RA. Spearman correlation analysis was used to explore the correlations of IL2RA expression and immune checkpoint genes.

A total of 747 DEGs were identified between low and high immune/stromal groups. Functional exploration revealed upregulated DEGs were associated with immune-related activities, whereas downregulated DEGs were involved in inflammatory-related activities. IL2RA was selected as the critical gene by overlapping the hub genes in PPI network and prognostic genes. Significantly, IL2RA expression was significantly elevated in PDAC and patients with higher IL2RA expression had worse prognoses. The immunological and oncogenic roles of IL2RA in PDAC were evidenced by GSEA. Furthermore, PDAC samples with high IL2RA expression exhibited increased immune infiltration and better immunotherapy responses. IL2RA expression was positively correlated with PDCD1, CD274, CTLA4, IDO1, TDO2, and TIGT.

Higher expression of IL2RA predicts worse survival outcomes and increased immune infiltration in PDAC. PDAC patients with high IL2RA expression might potentially benefit from immunotherapy.

## 1. Introduction

Pancreatic ductal adenocarcinoma (PDAC), the most common type of pancreatic cancer, is a highly malignant and aggressive solid tumor characterized by atypical symptoms, location concealment, rapid progression, and poor prognosis.^[1,2]^ In recent years, new chemotherapeutic drugs and surgical techniques have also made continuous progress for the treatment of PDAC.^[3]^ However, due to its early metastasis and chemoresistance, the 5-year survival rate of PDAC is no more than 10%.^[4]^ It is more regrettable that most patients have developed into advanced stages when they are diagnosed and lost the opportunity for surgical treatment.^[5]^ Recently, the development of tumor immunotherapy has completely revolutionized cancer treatment. At present, T cell receptor immunomodulators, immune checkpoint blockade (ICB), CAR-T cell therapy, and tumor vaccine are in the clinical trial stage of pancreatic cancer immunotherapy. However, their therapeutic effects on PDAC are not ideal as other types of malignancies.^[6]^

Tumor microenvironment (TME) is a complex environment composed of immune cells, stromal cells, adipocytes, fibroblasts, tumor vasculature, various cytokines and chemokines, and extracellular matrix molecules, etc.^[7]^ TME plays essential roles in regulating tumor progression and therapeutic response.^[8,9]^ Generally, TME is highly complex and dynamic. In the early stage of tumor, immune cells, and stromal components recruited and activated by tumor cells form a tumor-suppressive inflammatory microenvironment to hinder tumor development. However, with tumor progression, persistent tumor antigen stimulation and immune activation responses induce the exhaustion or remodeling of effector cells in TME, resulting in an immunosuppressive microenvironment. In this condition, immune cells in the TME lose anti-cancer functions and even promote the malignant progression of tumors.^[10]^ Herein, inducing tumor immunity and reversing the immune tolerance state of TME may be a promising therapeutic strategy for patients with PDAC. The immune activities of TME involves in complex molecular regulation mechanisms.

Stromal cells and immune cells of TME are important components mediating the progression and immunotherapeutic response of PDAC.^[11]^ In this study, we calculated the immune and stromal scores of 206 PDAC from GSE71729 using the “estimation of stromal and immune cells in malignant tumors using expression data” (ESTIMATE) method to evaluate the relative levels of infiltrating immune cells and stromal cells in the TME of PDAC. Through differential analysis, we screened out differentially expressed genes (DEGs) between low and high immune/stromal groups in the TME of PDAC. Following, gene ontology (GO) and Kyoto encyclopedia of genes and genomes (KEGG) pathway enrichment analyses were performed for upregulated DEGs and downregulated DEGs to investigate their underlying functions and involved pathways, respectively. Protein-protein interaction (PPI) network and Univariate Cox regression analysis was conducted and identified IL2RA as the key prognostic gene for PDAC. IL2RA (also termed as CD25), the alpha chain of the interleukin 2 receptor complex, is predominately expressed on the surface of mature T cell membrane and plays essential roles in immune regulation.^[12]^ Here, we confirmed IL2RA expression level was dramatically elevated and high IL2RA expression was positively correlated with poor prognosis of PDAC. Furthermore, we explored the relationships of IL2RA with clinical and immunological characteristics, immune checkpoint genes of PDAC. Taken together, we demonstrated IL2RA was a meaningful predictor for the prognosis and immune characteristics of PDAC, which might be used as the biomarker of prognosis and immune therapy assessment.

## 2. Materials and Methods

### 2.1. Data sources

The study is in accordance with relevant guidelines and regulations. The normalized gene expression profile and phenotype information of GSE71729 were downloaded from the gene expression omnibus (GEO) database (https://www.ncbi.nlm.nih.gov/geo/) using “GEOquery” R package. A total of 207 PDAC samples (145 primary and 61 metastatic tissues), and 134 control samples (46 pancreas and 88 distant site adjacent normal tissues) were extracted from GSE71729 for our analysis. The gene expression RNAseq-HTSeq-FPKM data and clinical data of GDC TCGA Pancreatic Cancer were obtained from UCSC Xena (https://xenabrowser.net/datapages/), which includes 4 normal adjacent tissues and 179 PDAC tissues. The detailed information of samples from GSE71729 and TCGA used in our study were listed in Table 1. In addition, the RNAseq data of 2921 normal tissues were downloaded from the genotype-tissue expression (GTEx) portal (https://gtexportal.org/home/), from which 167 normal pancreatic tissue were extracted for our analysis. All data used in the study were retrieved from free open-access public databases. Therefore, no ethical approval was required in this study. All informed consents of patients involved in this study were received prior to data collection in public databases.

**Table 1:** Clinicopathological characteristics of PDAC patients from GEO and TCGA.

Clinical characteristics	GSE71729 (n = 206)	TCGA-PAAD (n = 185)
Age at diagnosis (y)	-	
<=60		61 (32.97%)
>60		124 (67.03%)
Gender	-	
Male		102 55.14%)
Female		83 (44.86%)
Stromal subtype		-
low	57 (27.67%)	
normal	50 (24.27%)	
activated	99 (48.06%)	
Tumor subtype		-
Classifical	132 (64.08%)	
Basal	74 (35.92%)	
Stage	-	
I		21 (11.35%)
II		152 (82.17%)
III		4 (2.16%)
IV		5 2.70%)
NA		3 (1.62%)
T classification	-	
T1		7 (3.78%)
T2		24 (12.98%)
T3		148 (80%)
T4		4 (2.16%)
NA		2 (1.08%)
M classification		
M0	61 (29.61%)	85 (45.95%)
M1	145 (70.39%)	5 (2.70%)
MX	0 (0%)	95 (51.35%)
N classification	-	
N0		50 27.03%)
N1		130 (70.27%)
NX		4 (2.16%)
NA		1 (0.54%)

PDAC = pancreatic ductal adenocarcinoma, GEO = gene expression omnibus, TCGA = the cancer genome atlas.

### 2.2. Calculation of immunescore, stromalscore, and ESTIMATE score

ESTIMATE is a computational method proposed by Yoshihara et al in 2013.^[13]^ It utilizes gene expression data to calculate the immune/stromal scores of tumor tissues to evaluate the immune infiltration levels of TME. Here, we used ESTIMATE method by “estimate” R package to calculate the immune score and stromal score of each sample, which respectively represent the relative abundance of immune and stromal components in the TME of PDAC.

### 2.3. Identification of differentially expressed genes (DEGs)

A total of 206 PDAC samples of GSE71729 were divided into low and high immune/stromal groups according to the median value of immune/stromal scores, respectively. To identify DEGs between low and high immune/stromal groups, differential expression analysis was performed for the normalized expression data of GSE71729 using “limma” R package. Adjusted *P* value (Adj. *P*) <.05 and |log2foldchange| >.585 were set as the threshold. “pheatmap” and “ggplot2” R packages were respectively applied to visualize DEGs. Subsequently, we obtained the common upregulated and downregulated DEGs in high immune group and high stromal group through Venn diagrams by R package “VennDiagram”.

### 2.4. Functional enrichment analyses

R package of “clusterProlifer” was used to perform GO terms annotation and KEGG enrichment analyses for the upregulated or downregulated DEGs both in high immune group and high stromal group, respectively.^[14,15]^ Amongst, GO terms were composed of cellular component (CC), molecular function (MF), and biological process (BP). The results of GO and KEGG enrichment analyses were visualized by R package “ggplot2”. Only terms or pathways with *P* value <.05 and *q* value <0.05 were considered significantly enriched.

### 2.5. PPI network construction and Univariate Cox regression analysis

The interactions of common upregulated DEGs and downregulated DEGs were respectively obtained from the STRING database (https://string-db.org/). Nodes with interaction scores larger than 0.9 were used for PPI network construction by Cytoscape 3.8.2. In the PPI network, we extracted genes with the degree of the nodes ≥3 as hub genes. Besides, Univariate Cox analysis was applied to explore the relationships between the common DEGs and prognosis of PDAC patients. Genes with *P* < .05 were considered as prognostic genes of PDAC. Overlapping hub genes and prognostic genes, the key TME-related genes of PDAC were obtained.

### 2.6. Gene set enrichment analysis (GSEA)

In the molecular signatures database (MSigDB, http://www.gsea-msigdb.org/gsea/msigdb/index.jsp), KEGG gene sets of C2 and hallmark gene sets were applied as the target sets to perform GSEA using the software gsea-4.0.1. The gene expression data of GSE71729 was used for GSEA, and only gene sets with normalized *P *< .05 and false discovery rate (FDR) *q *< 0.06 were considered as significant.

### 2.7. Evaluation of the relationships between IL2RA expression and the immunological characteristics of PDAC

Anti-cancer immune response, also termed as the Cancer-Immunity Cycle, is composed of the following stepwise events: Release of cancer cell antigens (Steps 1), Cancer antigen presentation (Steps 2), Priming and activation (Step 3), Trafficking of immune cells to tumors (Steps 4), Infiltration of immune cells into tumors (Steps 5), Recognition of cancer cells by T cells (Steps 6), Killing of cancer cells (Steps 7).^[16]^ The signature gene sets of all these seven steps were downloaded from TIP (http://biocc.hrbmu.edu.cn/TIP/).^[17]^ Then the single-sample GSEA (ssGSEA) method was used to quantify the relative activities of anti-cancer immunity of 206 PDAC samples from GSE71729. Similarly, the relative abundance of tumor-infiltrating immune cells (TIICs) in the TME of these samples were calculated according to the gene sets of 28 types of immune cells obtained from the study of Charoentong.^[18]^ Besides, we evaluated the relative enrichment degrees of 21 gene signatures associated with the clinical response to the anti-PD-L1 agent atezolizumab by ssGSEA algorithm.^[19]^ Following, these 206 PDAC samples were divided into low-IL2RA and high-IL2RA groups based on the median value of IL2RA expression. Then Wilcoxon rank sum test was applied to compare the enrichment scores of anti-cancer immunity, TIICs, and immunotherapy response between low-IL2RA and high-IL2RA groups, with *P* < .05 as significantly different. Furthermore, the correlations of IL2RA expression with several immune checkpoint genes were analyzed through Spearman correlation analysis.

### 2.8. Statistical analysis

Statistical analysis was conduct by R language (version 3.6.3). Wilcoxon rank sum test (comparison between 2 groups) and Kruskal-Wallis rank sum test (comparison among three groups) were used to compare the difference of IL2RA expression in different groups. Kaplan–Meier survival analysis and log-rank test were conducted by “survival” and “survminer” R packages to analyze the prognostic value of IL2RA expression for PDAC patients, with automatically generated cutoff value of IL2RA expression. It was considered *P* < .05 to be statistically significant unless especially mentioned.

## 3. Results

### 3.1. Identification of DEGs based on immune scores and stromal scores of PDAC

**Figure 1.**
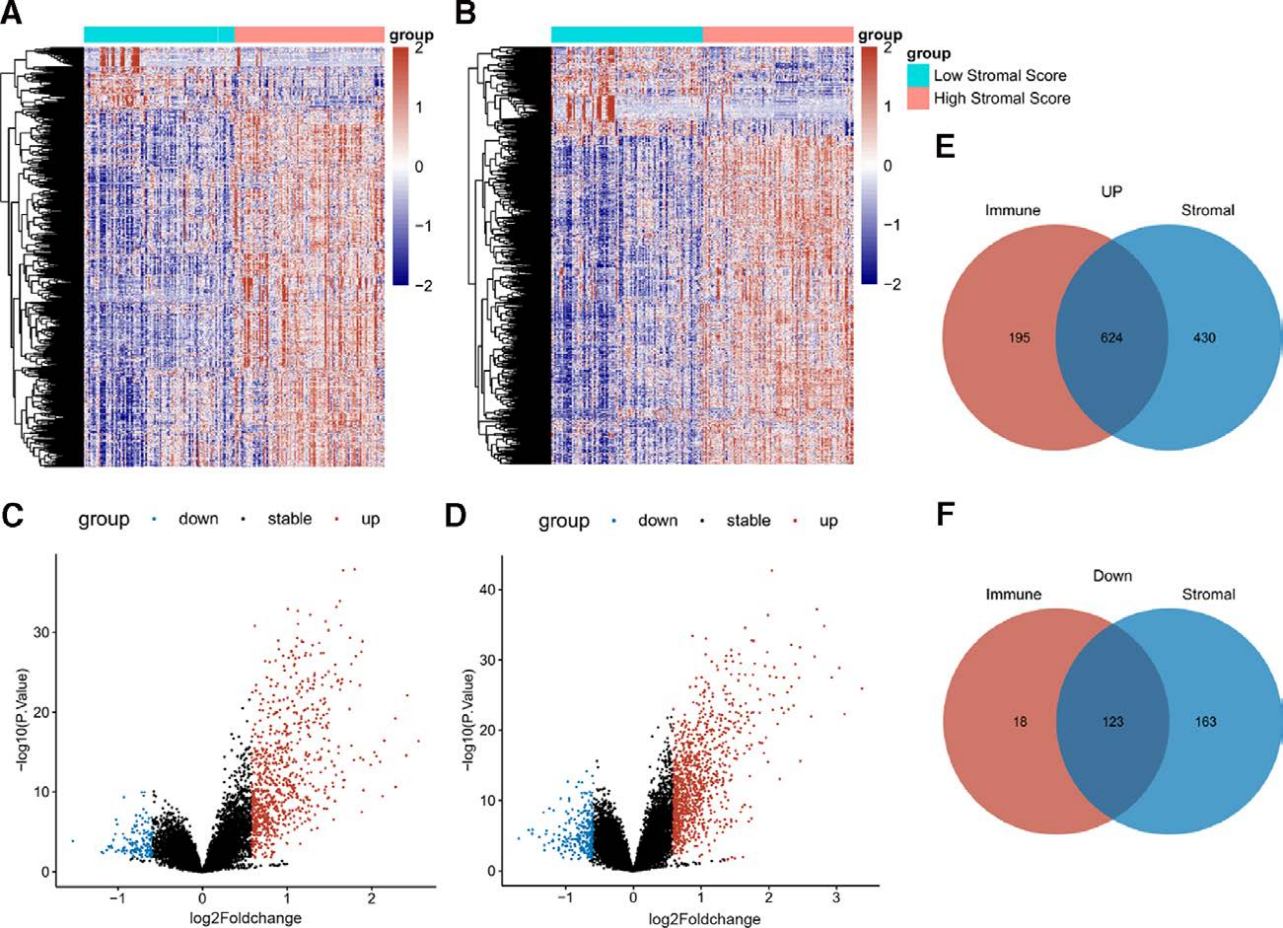
Identification of differentially expressed genes (DEGs) between low and high immune/stromal groups in PDAC. A. B, Heatmaps of DEGs between low immune/stromal and high immune/stromal groups, with blue representing relative low expression and red representing relative high expression. C. D, Volcano plots of the distribution of DEGs between low immune/stromal and high immune/stromal groups. Blue dots indicate significantly downregulared genes; red dots represent upregulated genes; black dots indicate genes with no significant difference. E. F, Intersection of upregulated (E) and downregulated genes (F) in high immune group and high stromal score by Venn diagram. PDAC = pancreatic ductal adenocarcinoma.

We calculated the immune scores and stromal scores of 206 PDAC samples from GSE71729. Then differential expression analysis was carried out for the normalized gene expression profiling of GSE71729, with the median value of immune/stromal score as the grouping criterion, identifying 960 (1340) DEGs between low and high immune (stromal) groups. Compared to low immune group, 819 genes were upregulated while 131 genes were downregulated in high immune group (Fig. 1A, C). Similarly, 1340 DEGs between low and high stromal group was composed of 1054 upregulated and 286 downregulated genes in high immune group (Fig. 1B, D). A list of 624 DEGs were both upregulated, whereas 123 DEGs were both downregulated in high immune group and high stromal group (Fig. 1E, F). Taken together, these 747 common DEGs have potential to be modulators of TME of PDAC.

### 3.2. GO terms and KEGG pathway enrichment analyses of the common DEGs

**Figure 2.**
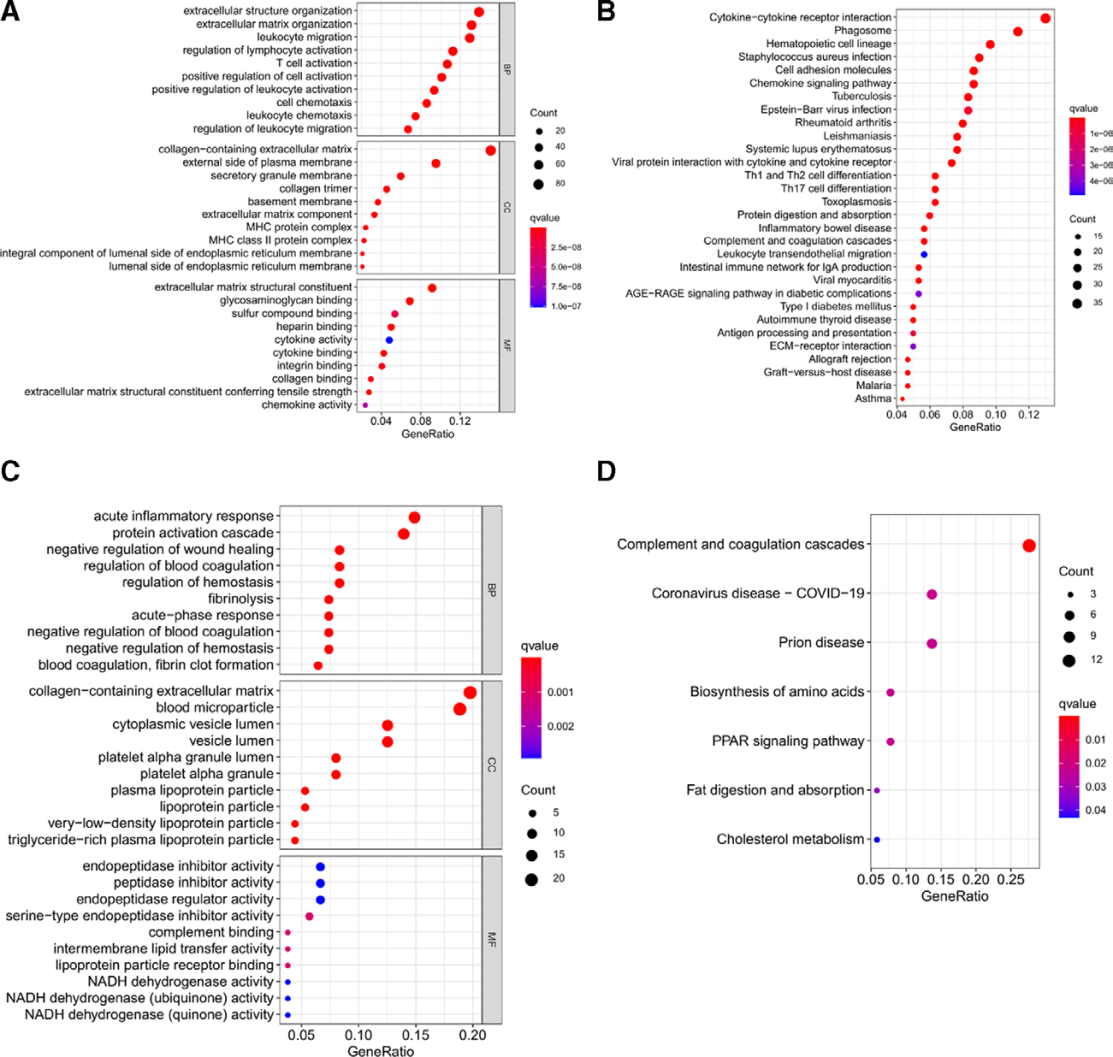
GO terms and KEGG pathway enrichment analyses of the common DEGs in high immune group and high stromal group. A. B, GO (A) and KEGG (B) enrichment analyses for 624 common upregulated DEGs. C. D, GO (C) and KEGG (D) enrichment analyses for 123 common downregulated DEGs. Counts: Number of genes enriched. DEGs = differentially expressed genes, GO = gene ontology, KEGG = Kyoto encyclopedia of genes and genomes.

To explore the underlying functions and pathways of the common upregulated and downregulated DEGs, we carried out GO and KEGG pathway enrichment analyses. The results of GO term annotation demonstrated these 626 upregulated DEGs were mainly enriched in extracellular structure organization, leukocyte migration, regulation of lymphocyte activation, T cell activation in BP; collagen-containing extracellular matrix, external side of plasma membrane, and secretory granule membrane in CC; extracellular matrix structural constituent, glycosaminoglycan binding, and sulfur compound binding in MF (Fig. 2A). The results of KEGG enrichment analysis revealed these 624 upregulated DEGs were significantly enriched in cytokine-cytokine receptor interaction, phagosome, hematopoietic cell lineage, cell adhesion molecules, chemokine signaling pathway, Th1 and Th2 cell differentiation, and Th17 cell differentiation, etc (Fig. 2B). While the results of GO term annotation demonstrated these 123 downregulated DEGs were mainly enriched in acute inflammatory response, protein activation cascade, and negative regulation of wound healing in BP; collagen-containing extracellular matrix, blood microparticle, and cytoplasmic vesicle lumen in CC; endopeptidase inhibitor activity, peptidase inhibitor activity, and complement binding in MF (Fig. 2C). The results of KEGG enrichment analysis revealed these 123 downregulated DEGs were significantly enriched in complement and coagulation cascades, coronavirus disease COVID 19, prion disease, biosynthesis of amino acids, PPAR signaling pathways, fat digestion and absorption, and cholesterol metabolism (Fig. 2D). Collectively, these common upregulated DEGs participated in various important immune-related functions and pathways. While these common downregulated DEGs were associated with inflammatory-related functions and pathways.

### 3.3. IL2RA was identified as the critical TME-related gene of PDAC

**Figure 3.**
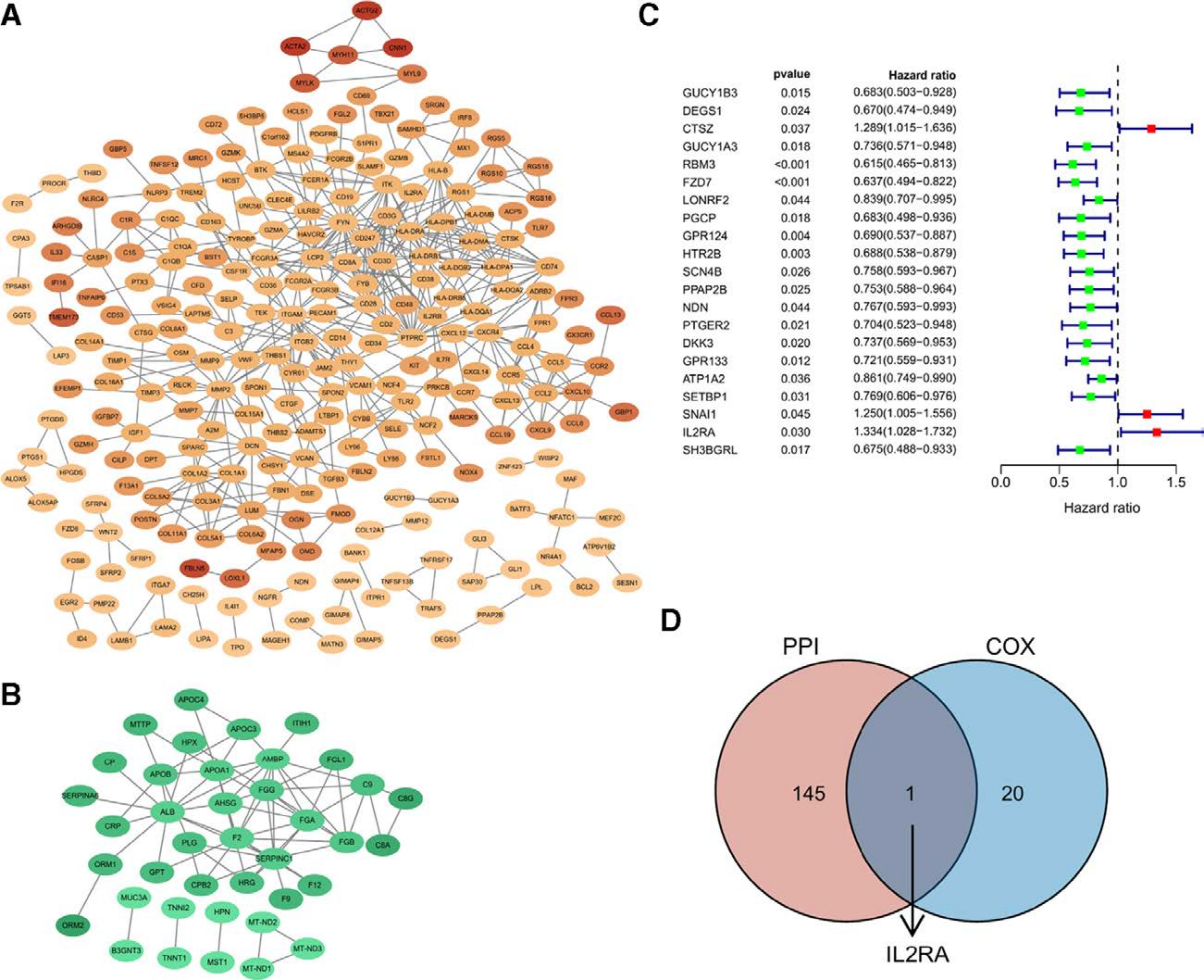
IL2RA was identified as the critical TME-related gene of PDAC. A, Six hundred twenty four common upregulated DEGs were used to construct PPI network. B, One hundred twenty three common downregulated DEGs were used to construct PPI network. C, Forest plot of 21 prognostic genes identified by Univariate cox analysis. D, Intersection of 146 genes with leading nodes in PPI network and 21 prognostic genes of PDAC by Venn diagram. DEGs = differentially expressed genes, PDAC = pancreatic ductal adenocarcinoma, PPI = protein-protein interaction, TME = tumor microenvironment.

To further investigate the relationships among the common 624 upregulated genes and 123 downregulated genes, their PPI were respectively obtained by STRING database and visualized by Cytoscape 3.8.2 (Fig. 3A and B). A total of 146 genes with the degree of nodes ≥3 was identified as hub genes (see Table S1, http://links.lww.com/MD/H522, Supplemental Digital Content, which illustrates genes with the degree of nodes ≥3 in PPI network). Following, Univariate Cox regression analysis was conducted for these 747 common DEGs and identified 21 prognostic genes of PDAC (Fig. 3C). Through taking the intersection of the 146 hub genes in PPI network and the 21 prognostic genes of PDAC, IL2RA was filtered out to be the critical TME-related gene of PDAC (Fig. 3D).

### 3.4. Identification of the clinical value of IL2RA in pancreatic cancer

**Figure 4.**
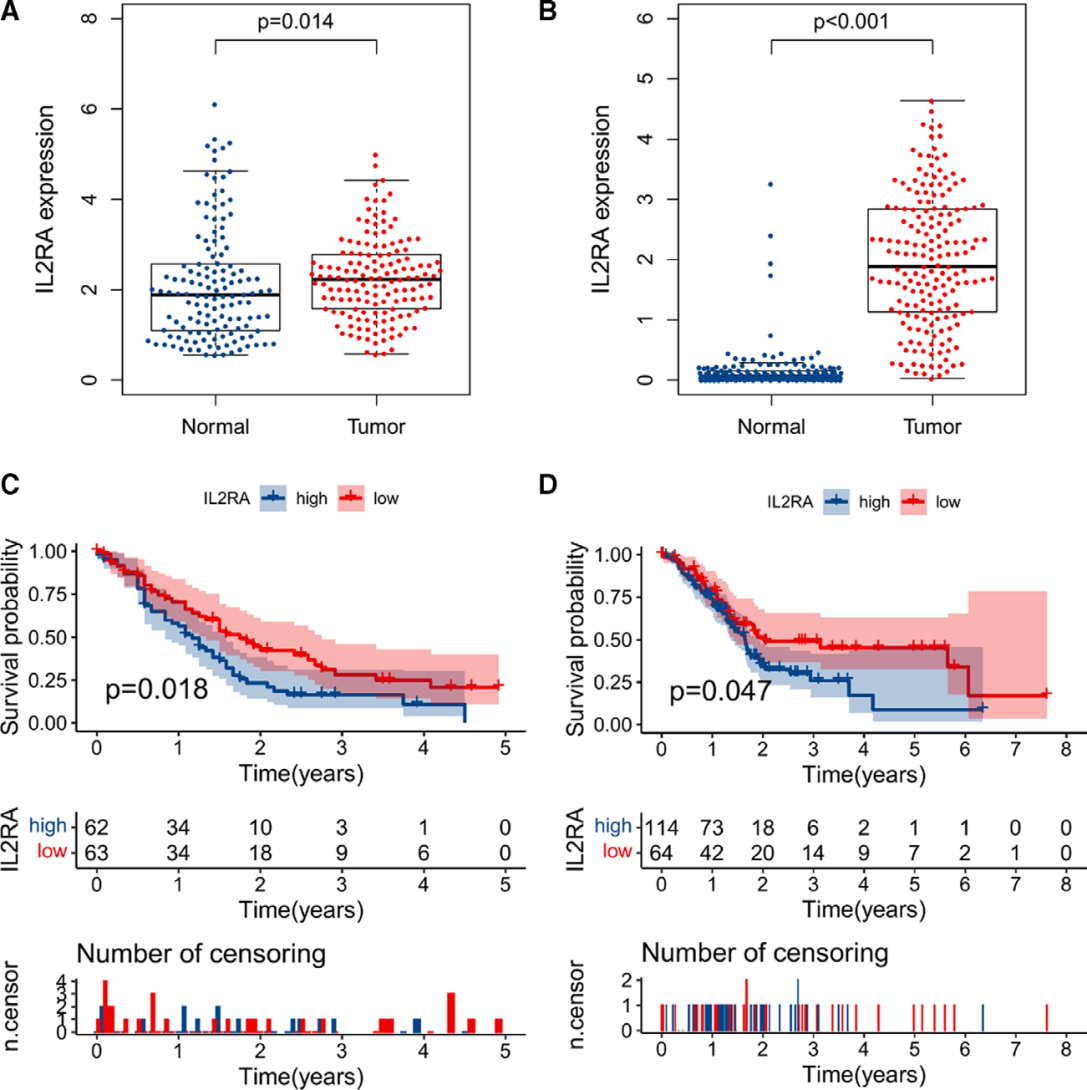
ILRA expression was significantly upregulated and associated with the prognosis of PDAC. A. B, Compared to normal tissues, IL2RA expression was significantly upregulated in PDAC tissues from GSE71729 (A) and GTEx + TCGA (B). C. D, Kaplan–Meier survival curve of overall survival of PDAC patients from GSE71729 (C) and TCGA (D) with low and high IL2RA groups. GTEx = genotype-tissue expression, PDAC = pancreatic ductal adenocarcinoma, TCGA = the cancer genome atlas.

**Figure 5.**
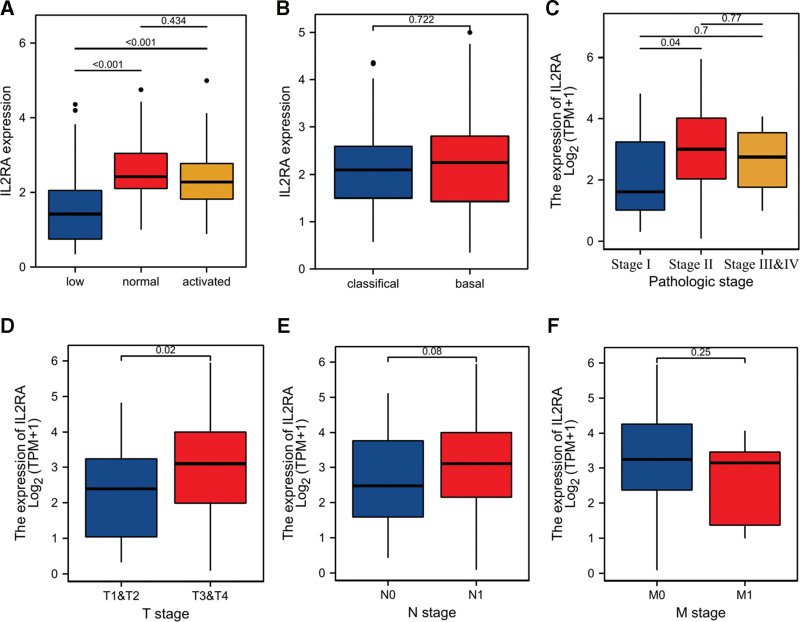
The correlations of IL2RA and clinical characteristic of PDAC patients. A. B, The difference of IL2RA expression between classical and basal types of PDAC (A), among low, normal, and activated immune (B) of PDAC from GSE71729. C-F, The difference of IL2RA expression among Stage I, Stage II, and Stage III & IV (C), T1 & T2, and T3 & T4 (D), N0 and N1 (E), M0 and M1 (F) of PDAC from TCGA. PDAC = pancreatic ductal adenocarcinoma.

Furthermore, we confirmed IL2RA was significantly upregulated in PDAC tissues compared to normal tissues from GSE71729, GTEx + TCGA (Fig. 4A, B). Importantly, Kaplan–Meier curve and log-rank test indicated PDAC patients with low IL2RA expression had longer survival time than those with high IL2RA expression (Fig. 4C, D). Moreover, we investigated the relationships between IL2RA expression and various clinical characteristics of PDAC patients. The results demonstrated IL2RA expression was significantly upregulated in normal and activated immune-status compared to low immune-status of PDAC patients from GSE71729 (Fig. 5A). However, IL2RA expression has no significant difference between classical and basal type of PDAC patients from GSE71729 (Fig. 5B). In PDAC patients from TCGA, we observed IL2RA expression was dramatically upregulated in Stage II compared to Stage I, T3, &T4 compared to T1 & T2, whereas no significant differences were observed between N0 and N1, M0, and M1 (Fig. 5C–F). These above results demonstrated that IL2RA had potential to be a meaningful biomarker for the prognosis of PDAC. In particularly, IL2RA might be an effective indicator for the immune status in the TME and play essential role in the progression of PDAC.

### 3.5. IL2RA expression was significantly correlated with immunological characteristics of PDAC

**Figure 6.**
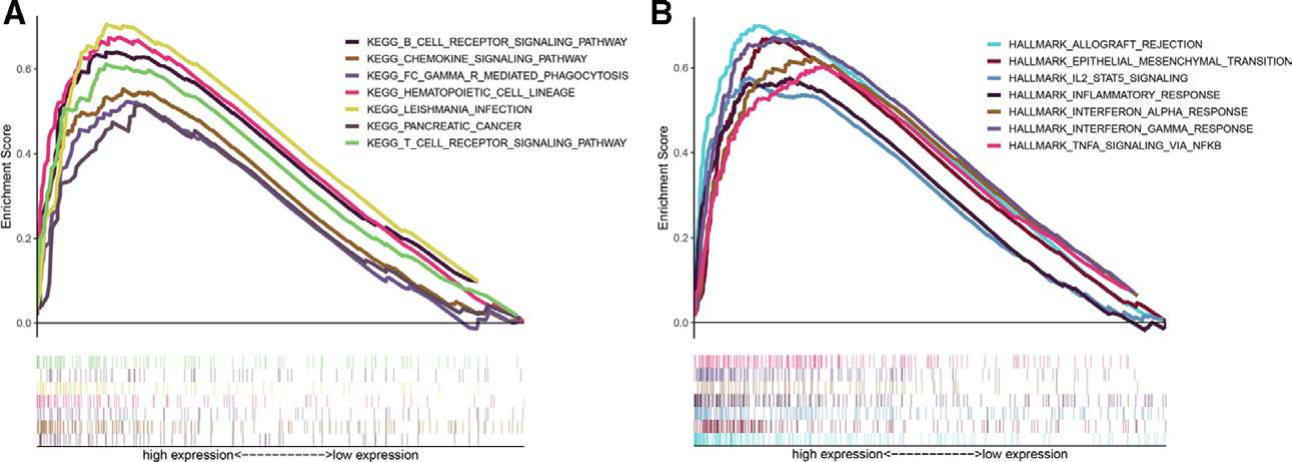
Exploration of molecular pathways and hallmarks associated with IL2RA in PDAC by GSEA. A, The enriched gene sets in KEGG by the samples with high expression of IL2RA. B, The enriched gene sets in HALLMARK by samples with high expression of IL2RA. GSEA = gene set enrichment analysis, KEGG = Kyoto encyclopedia of genes and genomes, PDAC = pancreatic ductal adenocarcinoma.

PDAC samples from GSE71729 were divided into low IL2RA and high IL2RA groups according to the median value of IL2RA expression. GSEA was implemented to explore immunological and oncogenic functions and pathways associated with IL2RA in PDAC. The results of KEGG enrichment indicated multiple immune-related pathways, including KEGG_B_CELL_RECEPTOR_SIGNALING_PATHWAY, KEGG_T_CELL_RECEPTOR_SIGNALING_PATHWAY, KEGG_CHEMOKINE_ SIGNALING_PATHWAY, and KEGG_PANCREATIC_CANCER, were significantly enriched in high IL2RA group (Fig. 6A). The results of hallmark gene sets enrichment also observed high expression of IL2RA mainly enriched inflammatory-related hallmarks (HALLMARK_ALLOGRAFT_ REJECTION, HALLMARK_INFLAMMATORY_RESPONSE, HALLMARK_INTERFERON_ ALPHA_RESPONSE, and HALLMARK_INTERFERON_ GAMMA_RESPONSE) and oncogenic hallmarks (HALLMARK_EPITHELIAL_MESENCHYMAL_ TRANSITION, HALLMARK_IL2_STAT5_SIGNALING, and HALLMARK_ TNFA_SIGNALING_VIA_NFKB) (Fig. 6B). The detailed enriched information was provided (see Table S2, http://links.lww.com/MD/H523, Supplemental Digital Content, which illustrates gene sets enriched by high IL2RA expression in PDAC). Collectively, these results further illustrated IL2RA was a critical oncogenic gene of PDAC and closely associated with the immune activities in the TME of PDAC.

**Figure 7.**
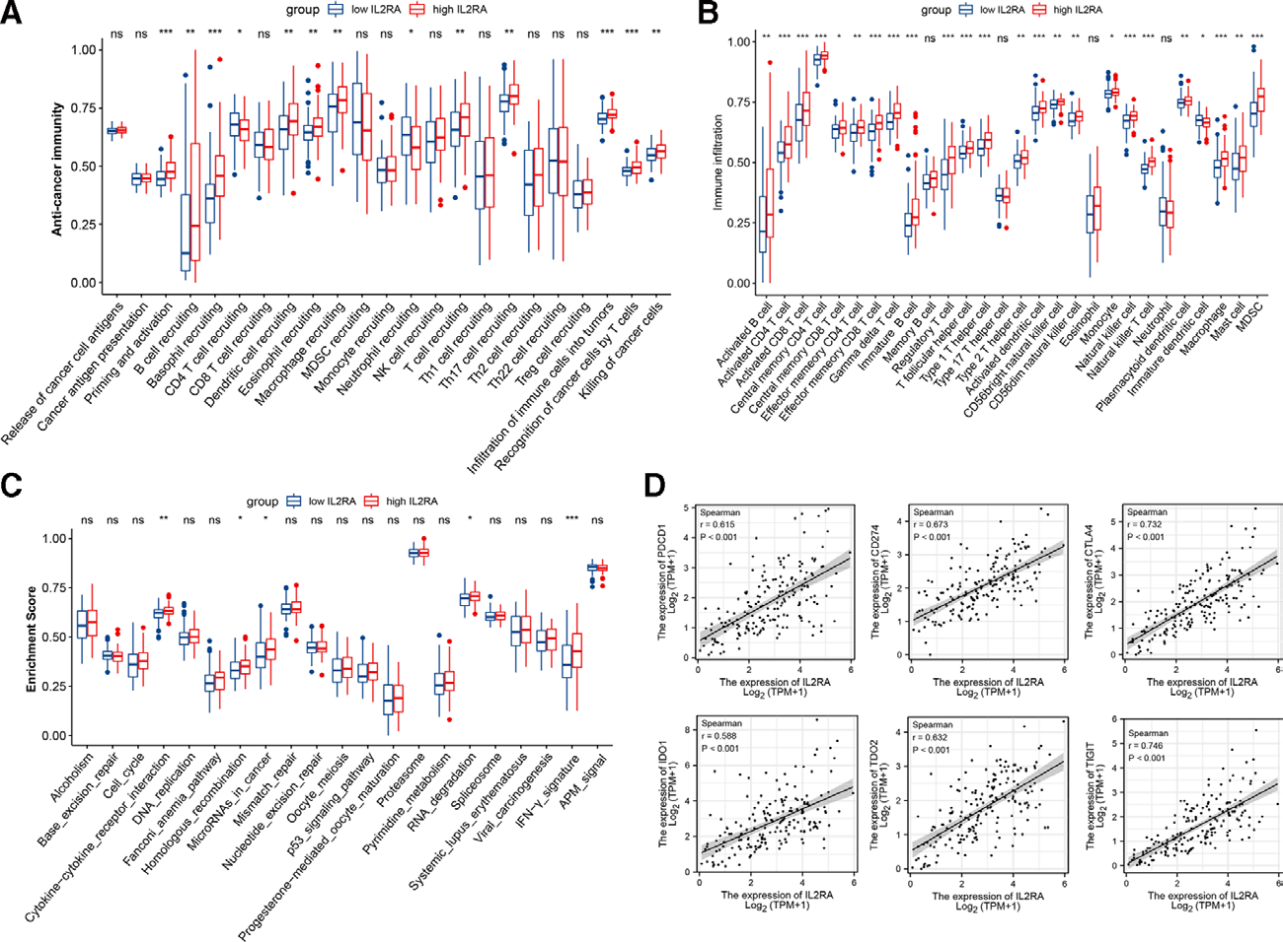
The relationships between IL2RA expression and immunological characteristics of PDAC samples. A, Activities of anti-cancer immunity between PDAC samples with low IL2RA and high IL2RA groups. B, The abundances of TIICs between PDAC samples with low IL2RA and high IL2RA groups. C, Difference in immunotherapy-predicted pathways between PDAC samples with low IL2RA and high IL2RA groups. D, Scatter plots show the positive correlations of IL2RA expression and the expression of several important immune checkpoint genes (PDCD1, CD274, CTLA4, IDO1, TDO2, and TIGIT).

Furthermore, we comprehensively analyzed the relationships between IL2RA and immune characteristics of PDAC. First, we compared the activities of anticancer immunity cycles between low IL2RA and high IL2RA groups. Significantly, the activities of Priming and activation, B cell recruiting, Basophil recruiting, CD4 T cell recruiting, dendritic cell recruiting, Eosinophil recruiting, Macrophage recruiting, Neutrophil recruiting, T cell recruiting, Th17 cell recruiting, Infiltration of immune cells into tumors, Recognition of cancer cells by T cells, and Killing of cancer cells were significantly higher in PDAC samples with high IL2RA (Fig. 7A). These results suggested that high IL2RA expression indicated activated anti-cancer immune responses of PDAC. Following, we compared the infiltration level of TIICs between PDAC samples with low IL2RA and high IL2RA groups. Likewise, the abundances of multiple TIICs, including activated B cells, activated CD4 T cells, activated CD8 T cells, etc, were significantly higher in PDAC samples with high IL2RA expression than those with low IL2RA expression (Fig. 7B). Meanwhile, we observed that pathways associated with the response of anti-PD-L1 agent atezolizumab, such as cytokine-cytokine receptor interaction, homologous recombination, MicroRNAs in cancer, RNA degradation, and IFN-γ signature, were significantly enriched in PDAC samples with high IL2RA expression (Fig. 7C). Moreover, Spearman correlation analysis revealed IL2RA expression was positively correlated with the expression of several classical immune checkpoint genes (PDCD1, CD274, CTLA4, IDO1, TDO2, and TIGT) (Fig. 7D). Collectively, these results supported that IL2RA might be a useful indicator for immune status and immunotherapy application of PDAC.

PDAC = pancreatic ductal adenocarcinoma, TIICs = tumor-infiltrating immune cells.

## 4. Discussion

PDAC is one of the most aggressive solid malignancies, with extremely high recurrence and mortality rates.^[3]^ In PDAC, malignant cells were surrounded with extracellular matrix, fibroblasts, endothelial cells, and immune cells, which constitute the TME of PDAC.^[20]^ Recently, increasing studies demonstrated that the TME plays a critical role in the progression and metastasis of PDAC.^[9,21,22]^ Dense desmoplasia and extensive immunosuppression were two major characteristics of the TME of PDAC, which contribute to cell proliferation, immune evasion, and failures of immunotherapy.^[23]^ Thus, it is of great significance to explore potential genes associated with immune activities in the TME of PDAC.

In our present study, we identified IL2RA as the key TME-related gene of PDAC. IL2RA was originally reported as one component of the high affinity heterotrimeric interleukin 2 (IL2) receptor on activated T cells.^[24]^ High IL2RA of haematological tumors has been well documented and was closely associated with poor prognosis.^[25,26]^ Additionally, IL2RA expression was reported to be elevated in several solid tumors, including head and neck cancers and epithelial ovarian cancer, which might acted as a stimulating driver for the proliferation of tumor cells through activating multiple oncogenic signaling pathways.^[27,28]^ Indeed, high IL2RA expression in activated circulating immune cells and Tregs has been used as a critical factor to exploit IL2 immunotherapies for treatment of tumors and autoimmune disease. The relative clinical safety and effectiveness of administering anti-IL2RA radiommunoconjugates and immunotoxins have been established and clinical trials of a novel anti-IL2RA targeted antibody drug conjugate are underway.^[29]^ However, the clinical values of IL2RA in PDAC are largely unclear. Here, we observed that IL2RA expression was significantly increased during the progression of PDAC and closely associated with the poor prognosis of PDAC patients. These phenomena illustrated the potential of IL2RA to be the prognostic biomarker for PDAC.

Through a series of bioinformatics analyses, we demonstrated the potential oncogenic and immunological roles of IL2RA in PDAC. Firstly, we explored the relationships between IL2RA expression and cancer-associated pathways and hallmarks in PDAC through GSEA. Interestingly, we found IL2RA might be involved in the modulation of B cell receptor signaling pathway, chemokine signaling pathway, T cell receptor signaling pathway, and inflammatory response. Pancreatic cancer of KEGG pathway and epithelial mesenchymal transition of hallmarks also might be activated by IL2RA. Following, we explored the relationships of IL2RA with anti-cancer immunity responses, abundances of TIICs, and immunotherapy-predicted pathways, demonstrating that high IL2RA expression in PDAC was associated with activated anti-cancer immune activities, TIICs, and immunotherapy responses. The above findings suggest that the TME of PDAC with high IL2RA levels tend to be “hot”, which indicated a great number of immune cells infiltrated into tumor, but were prevented to kill tumor cells by tumor-induced immunosuppression. Generally, “hot” tumors are more sensitive to immune checkpoint therapy.^[30]^ Currently, blocking immune checkpoints is one of the most promising immunotherapeutic strategies for cancer, which aim to reverse intra-tumoral T cell dysfunction and reactivate antitumor immunity.^[31]^ In PDAC, the expression of PD-L1 was proved to negatively correlated with the overall survival of patients. Co-treatment with anti-PD-L1 and anti-PD-1 significantly reduced tumor growth in mice subcutaneously injected with a murine PDAC cell line. However, the therapeutic effects of anti-PD-L1 monotherapy and anti-CTLA4 antibody Ipilimumab for PDAC patients were not satisfactory in clinical trials, which might attribute to the complexity of its TME.^[32–34]^ Here, we observed IL2RA expression was positively correlated with the expression of several major immune checkpoints, including PDCD1 (PD-1), CD274 (PD-L1), CTLA4, IDO1, TDO2, and TIGIT. Thus, we speculated PDAC with high expression of IL2RA might be more sensitive to immune checkpoint therapy.

Even though above findings, there exist several limitations in the study. We utilized public data to investigate the potential genes involved in the TME of PDAC and demonstrate the relationships between IL2RA expression and immunological characteristics in PDAC. However, we lack clinical data to validate these results. Besides, we did not detect the expression levels of different cells in the TME of PDAC, which require further effort in further experimental validation. At last, we did not systematically explore the underlying mechanisms of IL2RA involved in the activation of immune activities of PDAC.

In conclusion, our present study demonstrated that IL2RA has potential to be a biomarker to predict the prognosis and immune characteristics of PDAC, which indicated that IL2RA could be an indicator for personalized management and immunotherapy for PDAC patients.

## Author Contributions

L.-W.F. wrote the main manuscript text. L.-W.F., X.-Y.W., and L.-L.L. designed the study. Y.W. and S.-C.B. analyzed the data. L.-W.F., X.-Y.W.,Q.C., and W.-J.Y. prepared figures. All authors read and approved the final version of manuscript.

**Formal analysis:** Xinyu Wang.

**Investigation:** Xinyu Wang, Yue Wang, Wenjie Yang.

**Methodology:** Liwen Fan.

**Software:** Qing Chang.

**Supervision:** Linlin Liu.

**Validation:** Qing Chang.

**Writing – original draft:** Liwen Fan.

**Writing – review & editing:** Liwen Fan.

## Supplementary Material


