# Supplementary material for: IL2RA is a prognostic indicator and correlated with immune characteristics of pancreatic ductal adenocarcinoma
Source: Medicine (Baltimore). 2022 Oct 21;101(42):e30966. doi: 10.1097/MD.0000000000030966 (PMC9592409; doi:10.1097/MD.0000000000030966)
Supplement: Supplementary file 2 [file medi-101-e30966-s002.pdf]

Table S2. Gene sets enriched by high IL2RA expression in PDAC.

MSigDB collection	Gene set name	NES	NOM p-val	FDR q-val
	KEGG_T_CELL_RECEPTOR_SIGNALING_PATHWAY	1.976	0	0.074
	KEGG_B_CELL_RECEPTOR_SIGNALING_PATHWAY	1.938	0	0.057
**C2.cp.kegg.v7.4.symbols.g**	KEGG_LEISHMANIA_INFECTION	1.890	0	0.062
**mt**	KEGG_HEMATOPOIETIC_CELL_LINEAGE	1.882	0	0.052
high IL2RA expression	KEGG_CHEMOKINE_SIGNALING_PATHWAY	1.858	0	0.046
	KEGG_PANCREATIC_CANCER	1.854	0	0.042
	KEGG_FC_GAMMA_R_MEDIATED_PHAGOCYTOSIS	1.851	0	0.039
	HALLMARK_IL2_STAT5_SIGNALING	2.104	0	0.010
	HALLMARK_INTERFERON_GAMMA_RESPONSE	1.973	0.0058	0.021
	HALLMARK_ALLOGRAFT_REJECTION	1.998	0.0020	0.022
**h.all.v7.4.symbols.gmt**	HALLMARK_TNFA_SIGNALING_VIA_NFKB	1.854	0.0136	0.044
high IL2RA expression	HALLMARK_EPITHELIAL_MESENCHYMAL_TRANSITIO	1.773	0.0155	0.050
	N	1.777	0.0156	0.056
	HALLMARK_INFLAMMATORY_RESPONSE	1.746	0.0287	0.056
	HALLMARK_INTERFERON_ALPHA_RESPONSE			

PDAC, pancreatic ductal adenocarcinoma; NES: normalized enrichment score; NOM: normalized p-value; FDR: false discovery rate.
